# Inhibition of transient receptor potential melastatin 7 (TRPM7) protects against Schwann cell trans-dedifferentiation and proliferation during Wallerian degeneration

**DOI:** 10.1080/19768354.2020.1804445

**Published:** 2020-08-05

**Authors:** Young Hwa Kim, Sumin Lee, Hyejin Yang, Yoo Lim Chun, Dokyoung Kim, Seung Geun Yeo, Chan Park, Junyang Jung, Youngbuhm Huh

**Affiliations:** a Department of Anatomy and Neurobiology, College of Medicine, Kyung Hee University, Seoul, Korea; b Department of Biomedical Science, Graduation School, Kyung Hee University, Seoul, Korea; c Department of Otorhinolaryngology-Head and Neck Surgery, College of Medicine, Seoul, Korea

**Keywords:** Transient receptor potential melastatin 7 (TRPM7), trans-dedifferentiation, proliferation, Schwann cells, Wallerian degeneration

## Abstract

Irreversible peripheral neurodegenerative diseases such as diabetic peripheral neuropathy are becoming increasingly common due to rising rates of diabetes mellitus; however, no effective therapeutic treatments have been developed. One of main causes of irreversible peripheral neurodegenerative diseases is dysfunction in Schwann cells, which are neuroglia unique to the peripheral nervous system (PNS). Because homeostasis of calcium (Ca^2+^) and magnesium (Mg^2+^) is essential for Schwann cell dynamics, the regulation of these cations is important for controlling peripheral nerve degeneration and regeneration. Transient receptor potential melastatin 7 (TRPM7) is a non-selective ion (Ca^2+^ and Mg^2+^) channel that is expressed in Schwann cells. In the present study, we demonstrated in an *ex vivo* culture system that inhibition of TRPM7 during peripheral nerve degeneration (Wallerian degeneration) suppressed dedifferentiable or degenerative features (trans-dedifferentiation and proliferation) and conserved a differentiable feature of Schwann cells. Our results indicate that TRPM7 could be very useful as a molecular target for irreversible peripheral neurodegenerative diseases, facilitating discovery of new therapeutic methods for improving human health.

## Introduction

Wallerian degeneration (peripheral nerve degeneration) after peripheral nerve injury is a process by which degeneration advances toward the distal parts of peripheral nerves. The prominent morphological features of this process are axonal degradation and myelin fragmentation (Park et al. 2015). Schwann cells are important neuroglial cells that control these degeneration processes in the peripheral nervous system (PNS). Abnormal degeneration in the PNS dysregulates peripheral nerve regeneration and induces irreversible peripheral neurodegeneration, such as that seen in diabetic peripheral neuropathy. Thus, effective regulation of degeneration via Schwann cells during Wallerian degeneration is among the critical factors for maintaining normal PNS structures.

During Wallerian degeneration, Schwann cells exhibit morphologically, functionally, and biochemically distinct characteristics compared to normal, non-injured Schwann cells. Schwann cell status during degeneration is termed trans-dedifferentiation, as distinguished from differentiation, which occurs in normal Schwann cells (Jessen and Mirsky 2016). Trans-dedifferentiated Schwann cells function to repair injured structures in preparation for peripheral nerve regeneration. Another distinctive function of injured Schwann cells during Wallerian degeneration is proliferation; proliferated Schwann cells move into distal parts of the nerves and guide axonal regeneration to an innervation target. However, the process by which Schwann cell trans-dedifferentiation and proliferation are biologically controlled remains elusive.

Transient receptor potential (TRP) channels are non-selective ion channels that regulate homeostasis in biological systems by controlling calcium (Ca^2+^) and magnesium (Mg^2+^) influx (Ryazanova et al. 2010). The TRP subfamily consists of TRP ankyrin (TRPA), TRP canonical (TRPC), TRP melastatin (TRPM), TRP mucolipin (TRPML), TRP polycystin (TRPP), and TRP vanilloid (TRPV) (Busch et al., 2017). Several studies have shown that TRPs can alleviate abnormal conditions in the nervous system (Chen et al. 2015a, 2015b; Logu et al., 2017; Peters et al., 2012). In the PNS, Schwann cells are involved with TRPA1 and TRPM7, whereas during Wallerian degeneration, only TRPM7 expression increases (Logu et al., 2017; Chun et al. 2020). Therefore, control of TRPM7 expression could be an effective method for regulating Wallerian degeneration.

Peripheral neurodegenerative disease caused by diabetes mellitus, such as diabetic peripheral neuropathy, is increasingly common due to human lifestyle changes. However, therapeutic methods have not been discovered for treating diabetic peripheral neuropathy. In this study, using an *ex vivo* sciatic nerve culture system, we demonstrated that inhibition of TRPM7 protects against Schwann cell trans-dedifferentiation proliferation during peripheral nerve degeneration.

## Materials and methods

### Animals and ex vivo culture

Adult male 5-week C57BL/6 mice (Samtako, Osan, Republic of Korea) were used in this study. The sciatic nerve explant culture (*ex vivo* culture) was performed as previously described (Park et al, 2015). Briefly, under sterile surgical condition, the sciatic nerves were exposed 1.0 cm distal to the sciatic notch by blunt dissection, then harvested with a fine iris scissor (FST, Foster city, CA, USA). Sciatic nerves divided to 2–3 mm in length and then were incubated in Dulbecco Modified Eagle Medium (DMEM, Gibco-Invitrogen, Waltham, MA, USA) with 10% fetal bovine serum (FBS) and 1% penicillin/streptomycin (Gibco-Invitrogen, Waltham, MA, USA) at 37°C in a humidified atmosphere containing 5% CO_2_ with or without a TRPM7 inhibitor (carvacrol, Sigma-Aldrich, St. Louis, MO, USA; TG100-115, Selleck Chemicals, Houston, TX, USA). All surgical interventions were executed in accordance with the guidelines of animal experimentation established by The Korean Academy of Medical Science and were approved by the Kyung Hee University Committee on Animal Research [KHUASP(SE)-16-043-2]. We made an effort to minimize the number of animals used and their suffering.

### Immunofluorescent staining

Immunofluorescent staining was performed as previously described (Park et al, 2015). Briefly, the sciatic explants were fixed with 4% ice-cold paraformaldehyde (PFA) in phosphate-buffered saline (PBS) for 1 day at 4°C. Slides of teased or sectioned sciatic nerves were post-fixed of samples in 4% PFA for 10 min and then blocked with 5% bovine serum albumin in PBS with 0.3% Triton X-100 (PBST) for 1 hr at room temperature (RT). The slides were maintained overnight at 4°C with different markers such as S100 (Schwann cell marker; Sigma-Aldrich, St. Louis, MO, USA), lysosomal-associated membrane protein 1 (LAMP1, trans-dedifferentiation marker; Santa Cruz Biotechnology, Santa Cruz, CA, USA), p75 neurotrophin receptor (p75 NTR, trans-dedifferentiation marker; Santa Cruz Biotechnology, Santa Cruz, CA, USA), phosphorylated-extracellular signal-regulated kinase 1/2 (pERK1/2; Cell signaling, Beverly, MA, USA), Ki67 (proliferation marker; Abacm, Cambridge, UK) and EGR2/Krox20 (differentiation maker; Abacm, Cambridge, UK). On the following day, Alexa 488- or 594- conjugated secondary antibodies were added for 2 hr at RT. Nuclei of Schwann cells was visualized by 4′,6′-diamidino-2-phenylindole (DAPI) which was incubated for 10 min at RT after adding secondary antibodies. Imaging analyses were investigated with a LSM-700 confocal microscope (Carl Zeiss, Oberkochen, Germany).

### Western blot analysis

For Western blotting assay, sciatic nerve tissues were homogenized in ice-cold radioimmunoprecipitation assay buffer [RIPA; 25 mM Tris-HCl (pH 7.6), 150 mM NaCl, 1% Triton NP-40, 0.1% SDS (Thermo, Waltham, MA, USA)] with protease inhibitor mixture (Roche Molecular Biochemicals, Nutley, NJ, USA) and 1 mM Na_3_VO_4_ (Park et al, 2015). The lysate was then centrifuged for 15 min at 13,000 × *g* at 4°C. The supernatant was measured using Bradford assay (Bio-Rad, Hercules, CA, USA). After diluted with SDS loading buffer and boiled, separated protein in 10% polyacrylamide gels were then transferred onto nitrocellulose paper. The membrane were blocked with 5% (w/v) skimmed milk in Tris-saline solution (TBS, 20 mM Tros, 150 mM NaCl) with 0.05% Tween-20 (TBST) overnight at 4°C. After blocking the membrane, primary antibodies against p75 NTR, β-actin (Sigma-Aldrich, St. Louis, MO, USA), c-Jun (Santa Cruz Biotechnology, Santa Cruz, CA, USA), TRPM7 (Abacm, Cambridge, UK), p-ERK1/2 and p-p38 MAPK (Cell signaling, Beverly, MA, USA) were applied to the blot for 1 hr at RT and then secondary IgG-horseradish peroxidase-conjugated antibodies were applied for 1 hr at RT. Protein bands were visualized using an enhanced chemiluminescence system (GE Healthcare, Chicago, IL, USA). β-actin was used as a loading control.

### Statistical analysis

All data were analyzed using SPSS 21.0 software (IBM, Armonk, USA). All values are expressed as mean and standard error of the mean. Student’s *t*-test (non-paired two-tailed) were used to compare control (non-injured or non-cultured) and experimental groups or one-way ANOVA testing was used when multiple groups were compared. *p < 0.05, **p < 0.001, ***p < 0.0001 was considered significant and *p* > 0.05 was considered non-significant.

## Results

Carvacrol is a liquid phenolic monoterpenoid that is a major component of oil extracted from the flowers and leaves of *Origanum vulgare* and *Thymus vulgaris* (Angienda and Hill 2011). Previous studies have shown that carvacrol specifically decreases TRPM7 expression in injured Schwann cells (Chubanov et al. 2014; Chun et al. 2020). To investigate whether TRPM7 is involved in Schwann cell trans-dedifferentiation and proliferation during Wallerian degeneration, we used carvacrol (3 mM) as a specific inhibitor of TRPM7 expression in an *ex vivo* culture system (Chun et al. 2020).

### TRPM7 inhibition reduced expression of trans-dedifferentiation markers in ex vivo Schwann cells

**Figure 1.**
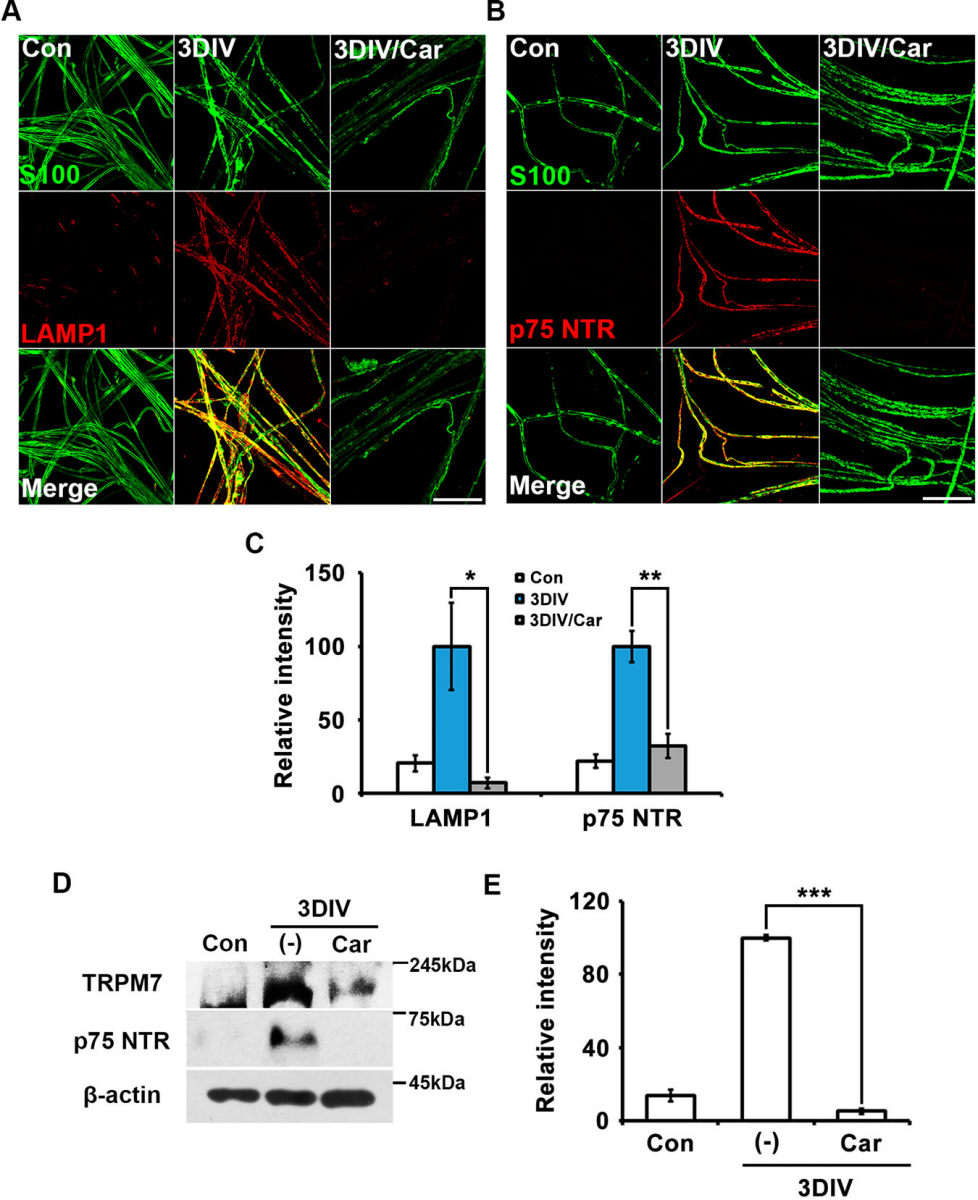
Inhibition of TRPM7 prevents trans-dedifferentiation of Schwann cells in *ex vivo* sciatic nerves. (A,B) Sciatic nerve fibers were immunostained with anti-S100 (green), anti-lysosomal-associated membrane protein (LAMP)-1 and anti-p75 neurotrophin receptor (p75NTR). Scale bar = 50 μm. (C) Relative intensities quantified the increase of LAMP1 and p75NTR expression in *ex vivo* trans-dedifferentiated Schwann cell (*n* = 3). (D) Western blot analysis showed protein expression of TRPM7 and p75NTR in *ex vivo* sciatic nerves. (E) Relative intensities indicated the effects of TRPM7 inhibition on trans-dedifferentiation (*n* = 9).

Trans-dedifferentiation during peripheral neurodegeneration is a unique characteristic of Schwann cells, through which they become similar to immature Schwann cells. To identify the effect of TRPM7 on Schwann cell trans-dedifferentiation, we immunostained for the lysosomal protein LAMP1 and nerve growth factor receptor p75 NTR as markers of Schwann cell trans-dedifferentiation (Park et al., 2015) using an *ex vivo* sciatic culture with or without treatment of carvacrol as a TRPM7 inhibitor. LAMP1 and p75NTR expression decreased in nerve fibers treated with the TRPM7 inhibitor compared to untreated degenerating fibers at 3 days *in vitro* (3DIV) (Figure 1A and B). Changes in LAMP1 and p75NTR expression with or without the TRPM7 inhibitor were observed in S100-positive cells acting as Schwann cell markers (Figure 1A and B). These results indicate that TRPM7 inhibition reduced the increase in LAMP1 and p75NTR expression in Schwann cells during Wallerian degeneration. Quantitative analysis also showed that TRPM7 inhibition significantly reduced LAMP1 and p75NTR expression at 3DIV (Figure 1C). Western blot analysis confirmed that carvacrol effectively inhibited TRPM7 expression at 3DIV compared with control (non-cultured or non-injured sciatic nerves) and that inhibition of TRPM7 suppressed p75NTR expression at 3DIV (Figure 1D and E). Together, these results suggest that TRPM7 was related to Schwann cell trans-dedifferentiation during Wallerian degeneration in an *ex vivo s*ystem.

**Figure 2.**
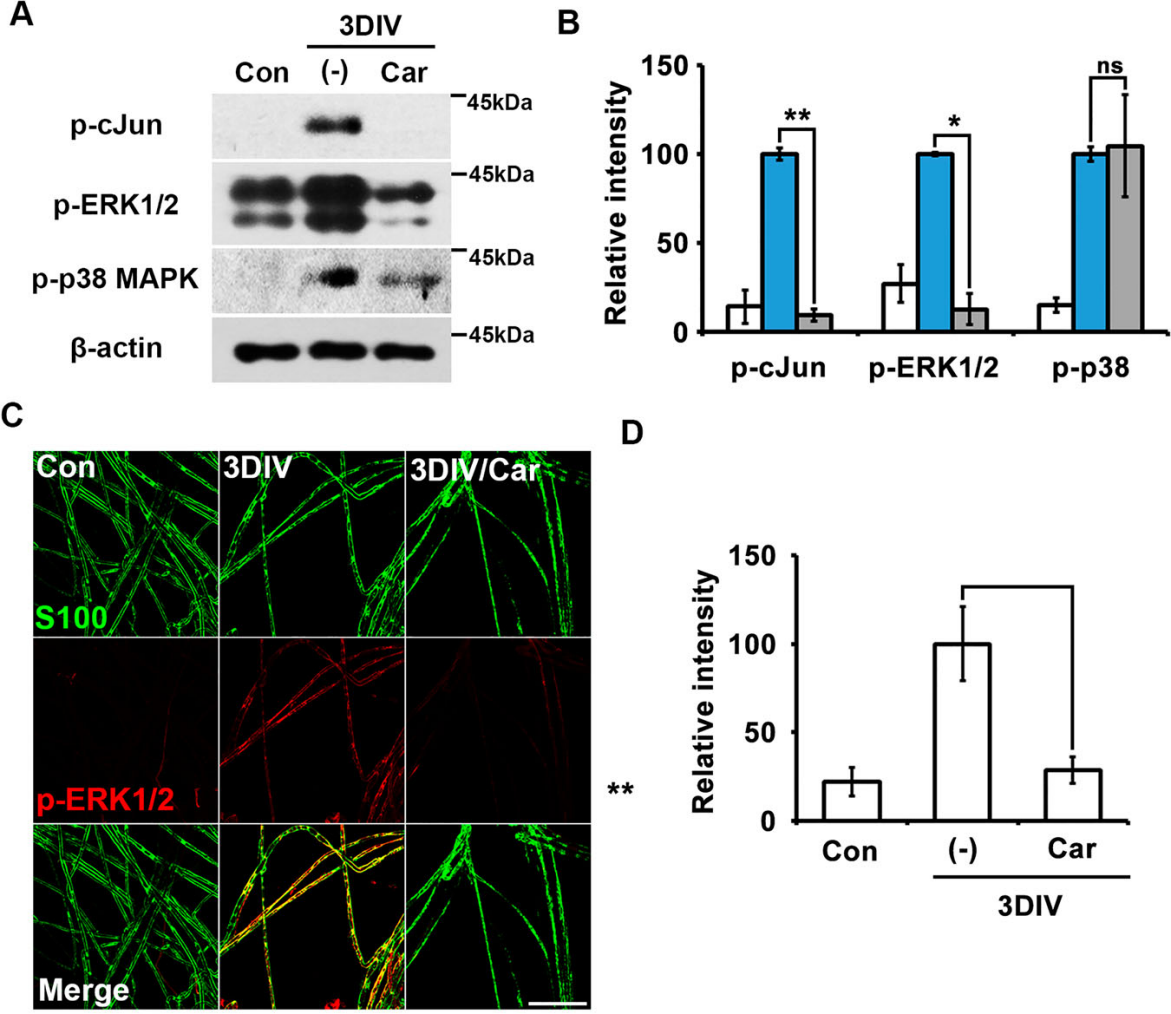
TRPM-regulated trans-dedifferentiation of Schwann cells involves in MAPK signaling pathway via a p38-MAPK-independent manner. (A) Western blot analysis showed protein expression of phosphorylated c-Jun (p-c-Jun), phosphorylated extracellular signal-regulated kinase (pERK)-1/2 and phosphorylated p38MAPK (p-p38MAPK) (B) Relative intensities quantified the expression patterns of p-c-jun, p-ERK1/2 and p-p38MAPK in *ex vivo* trans-dedifferentiated Schwann cell (*n* = 9). (C) Sciatic nerve fibers were immunostained with anti-S100 (green) and p-ERK-1/2 (red). Scale bar = 50 μm. (D) Relative intensities indicated the effect of TRPM7 inhibition on *ex vivo* Schwann cell trans-dedifferentiation (*n* = 3).

MAPK signaling is among the most important signaling pathways in trans-dedifferentiated Schwann cells (Parkinson et al. 2008). To access the relationship between TRPM7 and MAPK signaling, we performed Western blot analysis to quantify the expression of p-ERK1/2 (p44/p42 MAPK), p-p38-MAPK (MAPK14), and p-cJun (downstream of cJun-N-terminal kinase [JNK]-1/2). Our results showed that p-cJun and p-ERK1/2 expression decreased significantly after treatment with the TRPM7 inhibitor (Figure 2A and B). However, p-p38-MAPK expression was unaffected by TRPM7 inhibition (Figure 2A and B).

We also immunostained for pERK-1/2 in teased nerve fibers in the presence or absence of the TRPM7 inhibitor. pERK1/2 expression decreased in nerve fibers treated with the TRPM7 inhibitor compared to untreated degenerating fibers at 3DIV (Figure 2C and D). These results indicate that TRPM7 may regulate Schwann cell trans-dedifferentiation during the peripheral neurodegenerative process via the MAPK signaling pathway in a p38-MAPK-independent manner.

### TRPM7 inhibition reduced expression of cell proliferation markers in ex vivo Schwann cells

**Figure 3.**
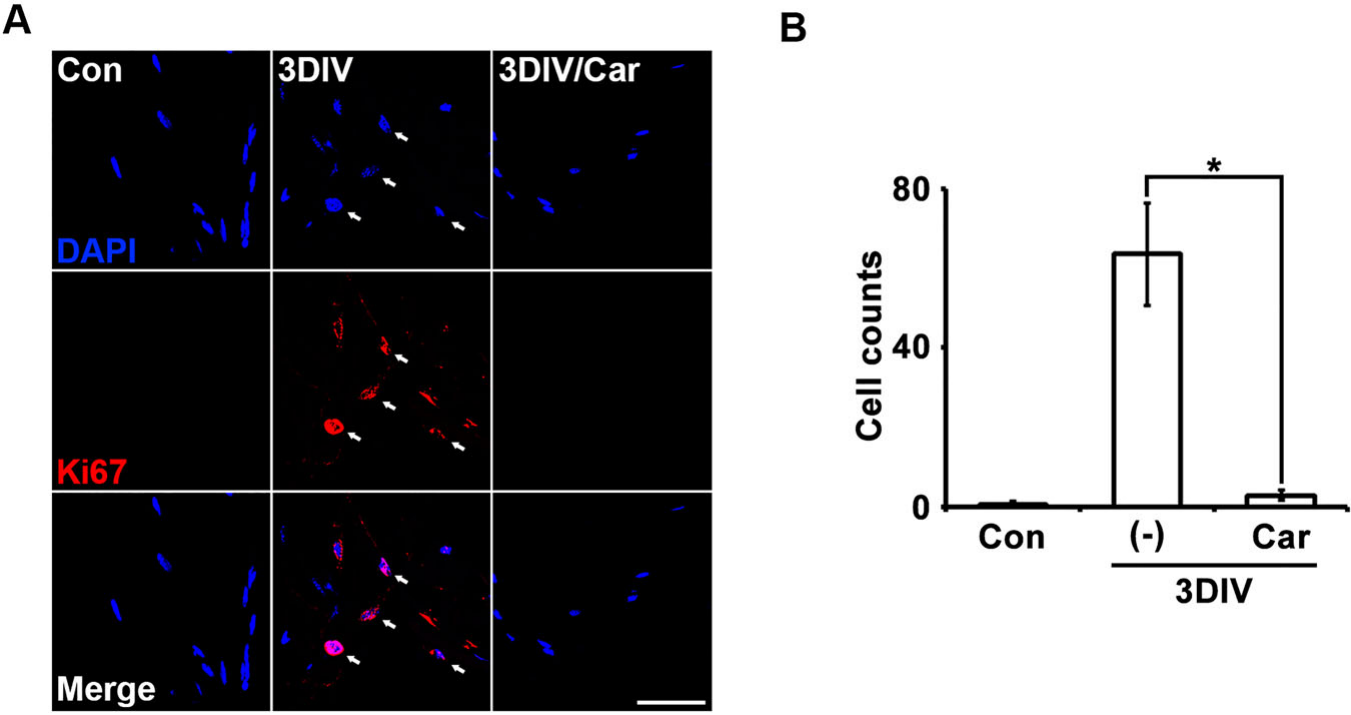
Inhibition of TRPM7 prevents Schwann cell proliferation in *ex vivo* sciatic nerves. (A) Sciatic nerve fibers were immunostained with anti-Ki67 (a marker for proliferation, red) and 4′,6-diamino-2-phenylindole (DAPI, blue). Scale bar = 50 μm. (B) Cell counts were the number of Ki67/DAPI double-positive cells (*n* = 3).

Degenerating Schwann cells proliferate rapidly, similar to immature Schwann cells. To confirm the relationship between TRPM7 and Schwann cell proliferation, we immunostained for Ki67, a marker of the overall active phase of the cell cycle in degenerating Schwann cells. At 3DIV, Ki67 expression in Schwann cells decreased on 4′,6-diamidino-2-phenylindole (DAPI)-positive nuclei after treatment with the TRPM inhibitor compared with untreated degenerating fibers (Figure 3A and B). These results indicate that TRPM7 may regulate Schwann cell proliferation during the peripheral neurodegenerative process.

### TRPM7 inhibition maintained expression of a differentiation marker in ex vivo Schwann cells

Trans-dedifferentiation is a dedifferentiable or degenerative feature of Schwann cells, unlike differentiation, which is a differentiable or normal feature of Schwann cells. To identify the molecular mechanism for TRPM7 inhibition to conserve differentiated Schwann cells, we performed immunostaining for Krox20, a critical transcription factor involved in the expression of myelin-related genes that represent Schwann cell differentiation (Woodhoo et al. 2009). Changes in Krox20 expression with or without the TRPM7 inhibitor were observed in S100-positive cells.

**Figure 4.**
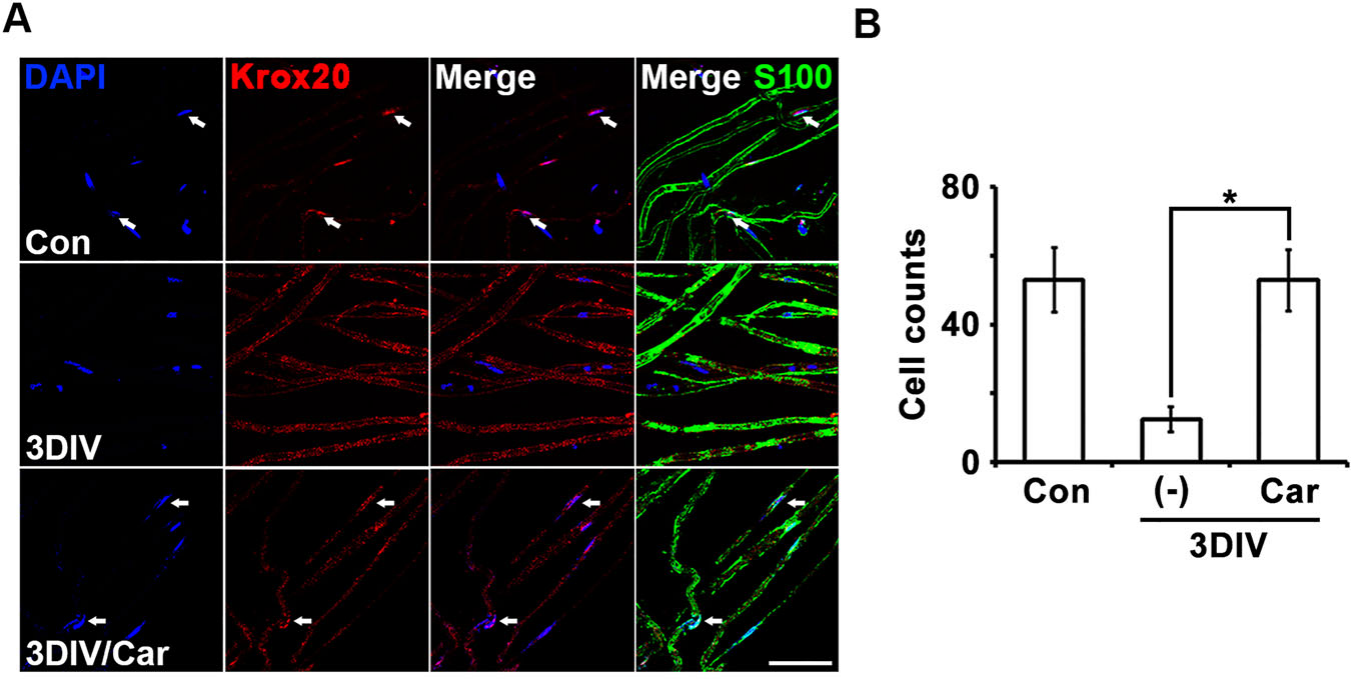
Inhibition of TRPM7 maintains Schwann cell differentiation in *ex vivo* sciatic nerves. (**A**) Sciatic nerve fibers were immunostained with anti-S100 (green), anti-Krox20 (red) and DAPI (blue). Arrows mean Krox20/DAPI double-positive cells. Scale bar = 50 μm. (**B**) Cell counts were the number of Krox20/DAPI double-positive cells per 200 DAPI cells (*n* = 3).

The number of Krox20/DAPI double-positive cells increased significantly in nerve fibers treated with the TRPM7 inhibitor at 3DIV compared with degenerating fibers (Figure 4A). Quantitative data also showed that TRPM inhibition maintained Krox20 expression in the *ex vivo* culture system. Therefore, TRPM7 inhibition may maintain Schwann cell differentiation during peripheral neurodegeneration via Krox20-dependent transcriptional regulation.

## Discussion

During trans-dedifferentiation, Schwann cells change their protein expression patterns to become similar to immature Schwann cells during development. Increased lysosomal activity is characteristic of trans-dedifferentiated Schwann cells (Park et al., 2015). In the present study, carvacrol-inhibited TRPM7 significantly decreased lysosomal activity (Figure 1). In a previous study, Ca^2+^ was found to significantly affect LAMP1 expression patterns in injured muscle tissue (Lennon et al. 2003). Although the direct effects of Ca^2+^, Mg^2+^, or both on p75NTR have not been reported, there are many examples in which Ca^2+^ dynamics are shown to be indirectly related to nerve growth factors. For example, decreased Ca^2+^ concentration decreased brain-derived growth factor levels in embryonic cortical neurons (Ghosh et al. 1994). In the present study, we demonstrated that TRPM7 inhibition effectively suppressed p75NTR expression in Schwann cells (Figure 1). Thus, TRPM7 may regulate LAMP1 and p75NTR expression in trans-dedifferentiated Schwann cells via the Ca^2+^ pathway, Mg^2+^ pathway, or both, either directly or indirectly.

The Ras/Raf/MEK/ERK pathway is among the most important signaling pathways during Schwann cell trans-dedifferentiation. Previous studies have shown that carvacrol, a TRPM7 inhibitor, reduces p-ERK1/2 expression (Chen et al. 2015b; Luo et al. 2016). Mg^2+^ or TRPM7 has been shown to affect p-ERK1/2 expression in several cell types (Zeng et al. 2015; Chen et al. 2015b; Liao et al. 2017). These reports are consistent with our result that TRPM7 inhibition decreased p-ERK levels in Schwann cells (Figure 2). Thus, TRPM7 may influence Mg^2+^- and Ca^2+^-triggered ERK signaling pathways during Schwann cell trans-dedifferentiation. C-Jun is an effector of JNK in the MAPK signaling pathway; in Schwann cells, c-Jun is a negative-regulatory transcriptional factor that maintains the demyelinated state of Schwann cells (Parkinson et al. 2008). A previous study reported that carvacrol attenuated p-JNK and c-Jun expression in lipopolysaccharide treated macrophages (Gholijani et al. 2016); p-JNK is also regulated by TRPM7 in astrocytes (Zeng et al. 2015). These results are consistent with our finding that TRPM7 inhibition decreased p-cJun expression (Figure 2A and B). However, TRPM7 inhibition did not suppress p-p38-MAPK in our research model (Figure 2A and B), consistent with a previous report that p-p38-MAPK levels are not regulated by TRPM7 (Zeng et al. 2015).

Degenerating Schwann cells increase their proliferation rate during peripheral neurodegeneration. In our study, inhibiting TRPM7 reduced the proliferation rate of Schwann cells (Figure 3). This finding is consistent with previous reports that carvacrol reduced the proliferation of prostate cancer cells dependent on the ERK pathway by inhibiting TRPM7 (Luo et al. 2016) and that TRPM7 affects glioma proliferation by regulating the G0–G1 cell cycle (Chen et al. 2016). Additionally, the Raf/Ras/MEK/ERK signaling pathway was shown to control Schwann cell proliferation at the G1 checkpoint (Lloyd et al. 1997); therefore, TRPM7 may regulate Schwann cell proliferation via this pathway. Together, these results indicate that TRPM7 is likely an essential component in the regulation of trans-dedifferentiation and proliferation of Schwann cells, depending on ERK1/2 and JNK/cJun, but not p38-MAPK, during the peripheral neurodegenerative process.

Our findings contradicted a previous report showing that Ca^2+^ boosts Krox20 expression in primary Schwann cells after treatment with dbcAMP (Figure 4; Kipanyula et al. 2013). However, because we used an *ex vivo* degeneration model whereas Kipanyula et al. (2013) used an *in vitro* development or regenerative model, comparisons between the two studies may not be meaningful.

On the other hand, in an experiment using TG100-115 (TG), another TRPM7 inhibitor (Song et al. 2017) for double-checking the effect of TRPM7, TG showed inhibitory effect on Schwann cell trans-dedifferentiation (Figure S1) via a concentration-dependent manner. Once again, we confirmed the TRPM7 handled Schwann cell trans-dedifferentiation and as a result, TRPM7 in Schwann cells could be a critical factor for regulating Wallerian degeneration. Additionally, our Wallerian degeneration model is *ex vivo* culture system using sciatic nerve explants, but not *in vitro* primary Schwann cell culture or the cell-line cultures. A peripheral nerve comprises three-layered connective membranes (epineurium, perineurium and endoneurium) and each membrane functions as a protective barrier against external environments. To increase the permeability of carvcrol into each connective membrane, in our *ex vivo* system, 3 mM of carvacrol was used and a concentration of less than 3 mM could not inhibit trans-dedifferentiation and proliferation of Schwann cells (data not shown).

Irreversible peripheral neurodegenerative diseases such as diabetic peripheral neuropathy are a worldwide therapeutic issue. The social and economic burden of neurodegenerative among aging populations disease treatment is increasing. Therefore, it is necessary to identify effective therapeutic targets for neurodegenerative diseases as quickly as possible. The results of the present study suggest that TRPM7, a divalent cation channel including Ca^2+^ and Mg^2+^, could be an effective molecular target for arresting or delaying peripheral neurodegenerative processes; its regulation could be an effective therapeutic method for treating irreversible peripheral neurodegenerative diseases.

## Supplementary Material

Supplemental Material
