# Supplementary figures and images for: Inhibition of transient receptor potential melastatin 7 (TRPM7) protects against Schwann cell trans-dedifferentiation and proliferation during Wallerian degeneration
Source: Anim Cells Syst (Seoul). 2020 Aug 5;24(4):189–96. doi: 10.1080/19768354.2020.1804445 (PMC7473164; doi:10.1080/19768354.2020.1804445)

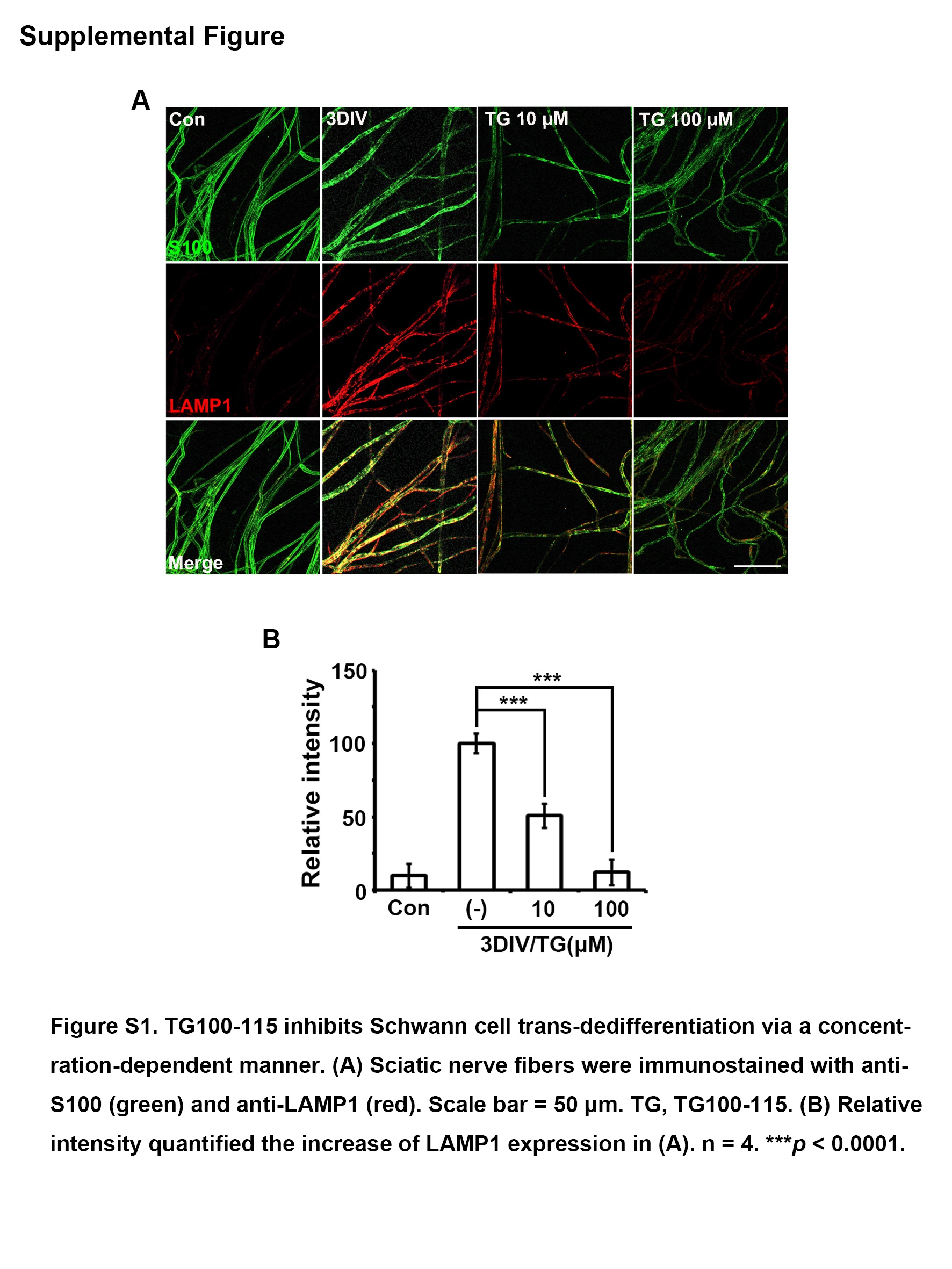

Supplement: Supplemental Material [file TACS_A_1804445_SM2671.jpg]
